# Co‐workers working from home and individual and team performance

**DOI:** 10.1111/ntwe.12153

**Published:** 2019-11-14

**Authors:** Tanja van der Lippe, Zoltán Lippényi

**Keywords:** working from home, performance, employees, co‐workers, organisations, multilevel

## Abstract

The number of firms supporting work from home has risen dramatically as advances in communication technology have fundamentally transformed the way humans cooperate. A growing literature addresses working from home, but focuses only on individual workers, overlooking potential influence of co‐worker engagement. Our aim is to study the influence of co‐workers working from home on individual and team performance. We use unique data from a large‐scale survey involving nine European countries, 259 establishments, 869 teams and 11,011 employees to show that the impact of working from home by co‐workers on performance is considerable and has remained hidden in past studies because they did not account for co‐worker effects. While working from home may be useful for some workers, it does bring issues for them as well. Specifically, we demonstrate that co‐workers working from home negatively impact employee performance. Moreover, team performance is worse when more co‐workers are working from home.

## Introduction

Working from home is firmly entrenched in modern working life and has become routine for many employees (Society of Human Resource Management, 2016; Vilhelmson and Thulin, 2016)*.* Against the backdrop of a growing number of dual‐earner couples, working from home was touted in the 1980s and 1990s as a cost‐effective option for improving employee performance by enhancing their work–life balance (Avery and Zabel, 2001). The practice even received institutional support, as both the US Congress and the European Union approved legislation supporting telecommuting arrangements for both private and public workers. Nowadays, the European Agreement on Telework improves the protection of people working from home and lays down rules to ensure they enjoy the same rights as other employees. In the United States, the Telework Enhancement Act helps employees enhance work–life effectiveness. Extensive prior research has focused on the influence of working from home on employee performance (Bailey and Kurland, 2002; Gajendran and Harrison, 2007; Martínez Sánchez *et al.*, 2007; De Menezes and Kelliher, 2011; Allen, Golden, and Shockley, 2015). The results of this research are, however, mixed: while some studies show that working from home leads to better performance (Vega, Anderson, and Kaplan, 2014; Allen *et al.*, 2015), others warn that working from home leads to social and professional isolation that hampers knowledge sharing (Crandall and Gao, 2005) and leads to the intensification of labour (Kelliher and Anderson, 2009; Felstead and Henseke, 2017). This paper provides a new explanation of why working from home influences performance, and the aim is to gain insight into the influence of co‐workers working from home on individual work performance. We argue that work performance is influenced not only by a particular worker’s working from home, but also by the extent to which his or her co‐workers work from home.

The impact of co‐workers remained hidden in past studies because their focus was restricted to the individual worker (Avery and Zabel, 2001) or to the organisation as a whole (Martínez Sánchez *et al.*, 2007), omitting the interplay between the individuals and their social environment within the context of their work. Studies of organisational behaviour are highlighting that the growing interdependence and complexity of tasks necessitates an analysis of how co‐workers influence organisational behaviour and outcomes (Chiaburu and Harrison, 2008; Vayre and Pignault, 2014).

In particular, our research meets a growing demand in the literature that behaviours of others in the workplace be studied to gain a better understanding of the individual behaviour of employees (Spreitzer, Cameron, and Garrett, 2017). We build on the notion that co‐workers cooperate and provide each other various forms of support that are essential to the functioning of the individual employee (Collins, Hislop, and Cartwright, 2016). In the context of teleworking, Windeler, Chudoba, and Sundrup (2017) show that maintaining a certain level of social interaction is important for employees’ functioning when they work from home. We extend the argument to conclude that teleworking impacts not only the performance of those employees who are engaged in it, but also their co‐workers, regardless of whether the co‐workers are engaged in telework. Disentangling the influences of individual and co‐worker teleworking is relevant because it provides management with a more complete assessment of the potential problems created by telework. To our knowledge, Golden (2007) is the only study to take the teleworking of co‐workers into account, but he focuses on co‐worker relations and analyses a single firm, and in his study, the percentage of co‐workers working from home is reported only by the individual employee.

In addition to analysing individual performance, this paper addresses the impact of working from home (as part of a team) on manager‐reported team‐level performance. The organisational literature on working from home often considers performance outcomes only at the level of individuals (De Menezes and Kelliher, 2011). However, multilevel theorists argue that performance of teams consists of more than simply adding up the individual efficiencies of each team member (Kozlowski and Klein, 2000: 17). Rather, team performance emerges out of the complex interplay between individual performances and the organisational processes of coordination, monitoring and control (DeNisi, 2000). Because managers are typically responsible for these processes, it is relevant to also address how managers evaluate the performance of teams on which many individuals are working from home.

We carried out a new, unique large‐scale workplace survey in nine countries to study how working from home affects employee and team‐level performance. Our survey spanned multiple industries in these countries, allowing us to study a wide variety of working from home contexts in organisations and work teams. As working from home implies that the emphasis has shifted more to output, as opposed to just being present, performance is a relevant indicator for study (Demerouti *et al.*, 2014). We focus specifically on home‐based telework. Home‐based telework refers to working at or from home during (at least part of) the employees’ contractual working hours (Felstead and Jewson, 2000; Sullivan, 2003; Peters and Van der Lippe, 2007). Employee work performance is evaluated in terms of the perceived proficiency with which an individual carried out the tasks specified in his or her job description (Koopmans *et al.*, 2014b). Our measure of co‐workers working from home is reported by the co‐workers themselves, and we also employ performance indicators reported by the employee and by the team manager, which help to minimise possible common method biases (Podsakoff *et al.*, 2003).

Working from home is only one of the flexible work arrangements present in 21st century organisations. These arrangements have both positive and negative outcomes. Cautions for example are raised about the potential costs of alternative scheduling strategies, such as increased need for managerial planning, and problems with interface and coverage with suppliers and customers (Baltes *et al.*, 1999). The literature also points to the divergences and paradoxes when it comes to telework outcomes (see, Boell *et al.*, 2016). Studies demonstrate positive outcomes such as a better work–life balance and less costs for the organisation. But negative outcomes are visible as well such as difficulties in sharing knowledge. In this paper, by studying the significance for performance of co‐workers working from home, we hope to engage with this literature and improve our understanding of the consequences of flexible work arrangements.

## Theory

### Co‐workers working from home and individual employee performance

To understand how co‐workers working from home influence individual performance, we first need to understand the role of working from home itself. The literature on work and labour processes lists both positive and negative aspects of working from home. Starting with the positive aspects, first, working from home should provide employees more opportunity to focus on their work tasks. When working away from the office, teleworkers are able to significantly reduce contact with other co‐workers. Indeed, research has shown that working from home is associated with fewer interruptions (Bailey and Kurland, 2002). Second, because nobody is physically monitoring the teleworking employee, teleworkers have greater discretion in how, under what conditions and sometimes when they can complete tasks (Kossek and Thompson, 2016). It increases employee flexibility over work demands (White *et al.*, 2003). More autonomy in the job is likely to be associated with more productivity (Vega *et al.*, 2014). Third, employees who can telework may be more willing to put in extra effort to reward their employer for the ‘favour’ of flexible work arrangements (Morgan, 2004; Kelliher and Anderson, 2009). In addition to the positive aspects, a number of negative aspects have also been identified. First, an important drawback of working from home is the decreased control by colleagues or the supervisor. This can be true for both the organisation and the employee. When someone’s work is poorly monitored, greater opportunity of work avoidance exists, but there might also be less feedback on potential errors. Team working might become a problem (Knights and McCabe, 2003). Team working is the will to govern but becomes more problematic when working from home. It might create uncertainties, tensions and resistance strategies by employees. Second, social and professional isolation might result once working at home (Kurland and Bailey, 1999; Crandall and Gao, 2005), which leads to less interpersonal networking, informal learning that enhances work‐related skills and mentoring from colleagues and supervisors. Being away from the office may also create a lack of visibility and increases teleworkers’ fear that being out of site limits opportunities for promotion, rewards and positive performance reviews (Cooper and Kurland, 2002).

Moving to co‐workers, they are likely to influence the performance of the employee in various ways. Individual attitudes and behaviours of workers are influenced by co‐workers in everyday ‘horizontal exchanges’ (Chiaburu and Harrison, 2008), including social exchange (Blau, 1964; Ten Brummelhuis, Haar and Van der Lippe, 2010), and reciprocity norms (Gouldner, 1960). Labour process literature on concertive control in teams indicates that shared norms developing in teams exert strong influence on workers (Barker, 1993; Sewell, 1998), and trust and shared experiences embedded in relationships among workers (Taskin and Edwards, 2007). ‘Tacit knowledge’ (Polanyi, 1958) about values, practices and systems of social exchange (Blau, 1964) is more likely to emerge in groups of workers, facilitating the functioning of group members. Group cohesiveness is positively related to performance, but when nobody is around, workers experience less cohesiveness (Cohen and Bailey, 1997). Following the job demands‐resources model (Bakker and Demerouti, 2007), trust and shared experiences improve performance by requiring less effort to maintain co‐worker transactions and by acting as a resource for knowledge and practical help. The exchange of critical information about how things in the organisation work makes tasks easier to execute by directly helping employees advance towards their work goals and by facilitating smoothing transactions with co‐workers (Chiaburu and Harrison, 2008). However, if such information is not exchanged between employees, this might affect their work intensification as they are not helped by others (Chung and Van der Lippe, 2018).

What do these insights suggest with regard to the influence of co‐workers working from home on individual employee performance? Consider a situation in which most of an employee’s co‐workers work from home. The negative aspects of this arrangement might outweigh the positive aspects, such as less ‘disturbance’ from co‐workers. As more co‐workers work from home, the employee’s interactions with them, including informal conversations and establishing shared experiences, are apt to become less common. Reciprocal norms in relationships are more difficult to establish if the worker has no opportunity to see his or her co‐workers. Furthermore, when many co‐workers work from home, it is less likely that someone will notice any problems the employee may have and provide support when needed (Golden, 2007). In other words, the lack of shared norms and information increases the likelihood of conflict and antagonism, which decreases the likelihood of positive co‐worker exchanges (Chiaburu and Harrison, 2008). Note that this is all of course dependent on the teleworking practice, that is how many days a week or hours a day a co‐worker works from home. Negative consequences of teleworking might surface only at a certain frequency of working from home. As it is an empirical question where such a threshold lies, we formulate a linear hypothesis but we take into account the possibility of a non‐linearity in our analyses. This leads to *Hypothesis 1: The more the co‐workers work from home, the weaker the performance of the individual employee.*

The few empirical results available support the existence of this negative aspect of co‐workers working from home. A higher prevalence of working from home by co‐workers results in workers being less satisfied with these co‐workers (Golden, 2007). Social disconnection has been shown to develop between employees working from home and office workers because working from home allows employees to distance themselves from work relationships at the office (Collins *et al.*, 2016). This might result in a less cohesive organisational culture. Although first results suggest a negative relation between colleagues working from home and work performance, working from home might not be the same as being absent from the workplace. Today’s working life includes many possibilities for establishing presence (or ‘face time’) at a distance, by digital media. Thus, being physically absent from work does not equal absenteeism in every sense of the word.

### Co‐workers working from home and team performance

So far, we have discussed the effect of co‐workers working from home on performance at the individual employee level, but what does this imply for the performance of the team? With respect to team performance, micro‐level behaviours lead to macro‐level outcomes in non‐additive fashion (Kozlowski and Klein, 2000). Above and beyond individual efficiency, costs of coordination, control and monitoring are also constituents of team performance (DeNisi, 2000). It is mostly managers who are responsible for coordinating, monitoring and controlling (i.e. correcting and motivating) the behaviour of team members in ‘vertical exchanges’ (Clear and Dickson, 2005). Direct managers’ assessments of team performance are thus relevant for understanding the impact of working from home on team efficiency. Managers directly experience the coordination and monitoring ‘costs’ of employees working from home, costs that factor into their assessment of team performance. First, coordination of a team involves integrating and aligning the actions of the team members (Rico *et al.*, 2010). Working from home makes coordination more complex. Consider a team with many members working from home, with 25 per cent of its members working more than half their time at home, 50 per cent working one day per week at home and 25 per cent working only at the office. This work environment is a complex setting, in which managers need to spend considerable effort organising how various types of employees cooperate and finish their work tasks. Interactions between co‐workers about tasks and work products must be scheduled or structured to compensate for the absence of chance encounters (Cooper and Kurland, 2002). Successfully managing this environment requires multitasking and a variety of skills and behaviours (Morris and Connaughton, 2017). Second, although abandoning direct managerial control and allowing employees’ discretion as to how they do their work is a growing trend in human resource practices, the lack of visibility of telecommuting employees can create many ambiguities. Direct managers may find it more difficult to assess employee productivity in terms of output and not ‘face time’ when employees work from home (Kossek and Thompson, 2016), as well as to motivate workers. Also, managers probably need to adopt new ways of monitoring employees (Sewell and Taskin, 2015), as teamwork is less possible to monitor for the manager. Lautsch, Kossek, and Eaton (2010) suggest that managers revert to alternative forms of control, such as encouraging information sharing and assistance with management of the work–family boundary, forms that are more ambiguous than traditional ones. So all in all, coordinating, monitoring and controlling processes become more complex and ambiguous in teleworking teams (Baruch, 2000), potentially decreasing the operational efficiency of the team. We therefore expect that team performance as evaluated by the team manager will be lower when team members are working from home, and this relationship will be directly proportional to the number of team members working from home. This leads to *Hypothesis 2: The more the team members are working from home, the lower the performance of the team.*

## Methods

### Sample

To test our hypotheses, we used the European Sustainable Workforce Survey (ESWS), which is a multi‐actor organisational survey conducted within organisations in Bulgaria, Finland, Germany, Hungary, the Netherlands, Portugal, Spain, Sweden and the UK (Van der Lippe *et al.*, 2016). These nine countries constitute different types of welfare regimes (Esping‐Andersen, 2009; Bäck‐Wiklund *et al.*, 2011). Although differences between these types are somewhat fluid, Finland and Sweden are typically categorised as socio‐democratic regimes, Germany and the Netherlands as conservative regimes, Spain and Portugal as Mediterranean regimes, the UK as a liberal regime, and Hungary and Bulgaria as post‐communist regimes. We used national business lists of organisations in the chosen industries in the country as our sampling frame. We chose establishments that belong to the six occupational industries under study and made a distinction between those with 20–99, 100–250 and 250+ employees. We randomly selected an organisation to approach from each sampling cell. The six industries are manufacturing, health care, higher education, transport, financial services and telecommunications. We selected them to reflect variation in the causes and types of investments in a sustainable workforce. These six industries vary therefore in the percentage of women working in the sector, the percentage of older employees, flexibility in contracting and the extent of technological development. If an establishment within a particular industry and size group refused to participate in the study, we used a matching strategy to include a new organisation within the same industry and within the same size category. In this paper, we use ‘organization’ to refer to the establishment. The organisations are in both the private and the public sector. After the organisation (often the HR director) agreed to participate, we contacted employees and their department managers at work and asked them to participate in an online or paper‐and‐pencil questionnaire. A total of 11,011 employees in 259 organisations participated in the survey. The participation rate at the organisation level varied from 5 to 20 per cent across countries. Given the difficulties involved in gaining access to organisations, non‐response is a relevant issue for the majority of studies that sample organisations (Van der Lippe, 2007). Once an organisation joined our research, the response rate was good: the within‐organisation response rate was 61 per cent for employees, 81 per cent among managers and almost 98 per cent for HR managers.

In our analyses of individual‐level performance, 2,372 respondents were removed due to item non‐responses as to one of the variables and two respondents were removed because they represented single observations within teams, resulting in an analytical sample size of 8,637 workers from 828 teams and 257 establishments. In our team‐level analyses, 152 teams were removed due to an item missing from one of the team‐level variables, 35 were removed because they were based on single observations of employees within teams, and 165 were removed as there was no intra‐organisational variation as to the dependent variable (productivity evaluation by managers). However, the results remain the same when including these teams. The analytical sample size at the team level is 516 teams from 153 establishments. Appendix 1 shows the country and industry composition of the analytical sample.

### Measures

#### Dependent variables

We measured performance based on the perceived efficiency in performing job tasks as reported by the employee. We used the task performance scale of the Individual Work Performance Survey battery, developed to produce comparable measures of self‐evaluated performance across different types of jobs (Koopmans *et al.*, 2012; Koopmans *et al.*, 2014a). The scale is characterised by good psychometric properties as well as invariance to country context and job type (Koopmans *et al.*, 2014b). Task performance is measured by five items on a five‐point Likert scale. Examples include: ‘I was able to plan my work so that I finished on time’ and ‘I was able to do my work efficiently’. The Cronbach alpha of the task performance battery is 0.85. We constructed the individual‐level performance by summing up the items. In addition to the employees’ subjective measurements, our study measures team performance by asking the direct manager, ‘How would you rate your team’s labor productivity?’ Using a four‐point Likert scale, team managers could choose between ‘very good’, ‘good’, ‘neither good nor bad’ and ‘rather poor’. We later merged the last two categories since few managers reported poor performance, and reversed the Likert scale numbers so that a high score implies better performance.

#### Independent variables

We have three independent variables, working from home by the individual employee, working from home by team co‐workers and working from home by the whole team. We measured individual employees working from home by the regularity of their doing so, following the measurement strategy recommended by Allen *et al. *(2015). The survey included the following question: ‘In the past 12 months, how often have you worked at home during normal working hours? Exclude overtime’. The response categories are (1) never or almost never, (2) <1 day a month, (3) <1 day a week, (4) 1 day a week, (5) 2 days a week, (6) 3 days a week and (7) 4 or 5 days a week. We included this measure as a categorical variable, but collapsed categories 5–7 into one category (‘working from home more than one day per week’). This is done because the percentages in the separate categories 5 and 7 were very low.

To measure working from home by team co‐workers, we calculated the proportion of regular teleworkers in the respondent’s team (excluding the respondent). We considered workers who work at home at least one day a month (category 3–7) as regular teleworkers. To assess the robustness of choosing this as threshold for regular teleworking, we estimated models with a measure of the proportion of workers in the team who work from home at least once a week. The size, significance and substantive conclusions do not change across the different specifications.

Finally, for working from home by the whole team, we started by recoding the original question to average hours per week for each worker: never or almost never as 0 hours, <1 day a month to 0.8 hours, <1 day a week to 4 hours, 1 day a week to 8 hours, 2 days a week to 16 hours, 3 days a week to 24 hours and 4–5 days a week to 36 hours. Subsequently, we took the average teleworking hours within each team and created the following categories: (1) no working from home, (2) <1 hour on average, (3) <4 hours on average, (4) between 4 and 8 hours on average and (5) more than 8 hours on average.

We do not expect the common method variance (CMV) to bias our estimates of the effect of individual employees and co‐workers working from home for two reasons. First, a respondent’s own working from home is measured objectively by asking the number of days worked from home rather than by a subjective evaluation of working from home frequency, which largely eliminates cognitive and information‐processing biases in its substantive association with performance (Glick, Jenkins, and Gupta, 1986), and second, the independent variable of working from home by co‐workers is constructed from other reports and its effect on performance is unlikely to be biased by method effects (see Podsakoff *et al.*, 2003).

#### Control variables

We control for a number of variables indicated in the literature as influencing employee performance. At the employee level, these include employees’ self‐reported level of job autonomy (measured by the sum of four (five‐point) Likert scale items (α = 0.86), taken with slight modifications from the job control inventory of Karasek, 1979); job satisfaction (measured by a single ten‐point scale item); organisational commitment (measured by the sum of four (five‐point) Likert scale items (α = 0.84), taken with slight modifications from the value commitment battery of Angle and Perry, 1981); physical job demands (measured by the sum of four (five‐point) Likert scale items (α = 0.75), taken with slight modifications from the Job Content Questionnaire of Karasek *et al.*, 1998); occupation (two‐digit ISCO), organisational tenure and tenure squared; part‐time versus full‐time work; contract type (permanent or temporary job); hourly wage; commuting time to work; years of education; presence of a partner at home and a youngest child younger than 16; and gender (1 = female) (Van der Lippe, 1994). We also include survey mode (paper vs. online) as a control variable. At the team level, we control for the proportion of permanent employees, part‐time employees, women, employees who have a partner and workers who are parents, plus commuting time, years of education and the average level of self‐reported autonomy, job satisfaction and employee commitment. Furthermore, we control for the size of the team, whether it was a team with a core or supporting function, the industry and the country. Descriptive statistics of all variables can be found in Table 1.

**Table 1:** Descriptive statistics

	Mean	SD	Min	Max
*Individual‐level analysis* a				
Performance	18.83	3.87	5	25
Working from home—never or almost never	0.71			
Working from home—<1 day a month	0.11			
Working from home—<1 day a week	0.07			
Working from home—1 day a week	0.06			
Working from home—more than 1 day a week	0.06			
Proportion of co‐workers WFH	0.19	0.25	0	1
Job autonomy	14.99	3.31	4	20
Job satisfaction	7.02	1.91	1	10
Organisational commitment	15.24	2.98	4	20
Job demands	13.48	2.66	4	20
Tenure in years	10.59	9.79	0.08	52
Permanent worker	0.89			
Part‐time worker	0.22			
Hourly wage^b^	12.11	10.49	0.01	339.93
Commuting time in hours	0.54	0.38	0.00	9
Years of education	14.33	2.24	6	20
Female	0.56			
Living with minor child at home	0.37			
Living with partner	0.74			
Average job autonomy—co‐workers	15.03	1.75	7	20
Average job satisfaction—co‐workers	7.02	0.88	1	10
Average commitment—co‐workers	15.23	1.47	6	20
Average job demands—co‐workers	13.49	1.30	8	20
Average tenure—co‐workers	10.72	5.97	0.17	44.00
Proportion of co‐workers with permanent contract	0.89	0.17	0	1
Proportion of co‐workers part time	0.22	0.26	0	1
Average hourly wage—co‐workers	12.07	5.92	2.16	174.03
Average commuting time—co‐workers	0.55	0.19	0.03	1.73
Average years of education—co‐workers	14.28	1.65	10	20
Proportion of co‐workers—female	0.56	0.30	0	1
Proportion of co‐workers with minor child	0.37	0.18	0	1
Proportion of co‐workers with partner	0.74	0.16	0	1
Paper‐and‐pencil survey mode (ref: online)	0.24		0	1
*Team‐level analysis*				
Performance not good^c^	0.13		0	1
Performance good^c^	0.52		0	1
Performance very good^c^	0.35		0	1
Working from home team—never	0.25		0	1
Working from home team—<1 hour on average	0.32		0	1
Working from home team—<4 hours on average	0.25		0	1
Working from home team—between 4 and 8 hours on average	0.10		0	1
Working from home team—more than 8 hours on average	0.07		0	1
Average job autonomy	15.07	1.83	8	20
Average job satisfaction	7.01	1.00	2	10
Average organisational commitment	15.41	1.58	9.50	20
Average job demands	13.47	1.45	9.33	19
Average tenure in years	9.86	6.15	0.24	32.80
Proportion of permanent workers	0.88	0.18	0	1
Proportion of part‐time workers	0.19	0.25	0	1
Average hourly wage^b^	11.76	4.93	3.09	33.92
Average commuting time in hours	0.55	0.21	0.17	1.49
Average years of education	14.22	1.55	10.73	20
Proportion of female workers	0.55	0.31	0	1
Proportion of workers with minor child	0.37	0.21	0	1
Proportion of workers living with partner	0.74	0.19	0	1
Size of team (no of workers)	13.31	12.18	1	83
Core function team (ref: supporting)	0.74		0	1

Notes

In individual‐level analyses, the number of workers is 8,637, the number of teams is 828, and the number of establishments is 257. In team‐level analyses, the total number of teams is 516, and the number of establishments is 153.

a Indicators representing two‐digit ISCO 2008 occupational categories not included here but are controlled for in individual‐level analyses.

b In Euros, harmonised to the Eurostat price level index based on household final consumption expenditures per country.

c Reported by team manager.

### Analytical strategy

When analysing individual‐level performance, we used linear regression including fixed effects for team to control for team‐level and establishment‐level confounders, and controls for the team composition.

In the model for manager‐evaluated team‐level performance, we used ordinal logistic regressions, adding fixed effects for the establishment level and additional controls for team composition, the number of employees on the team and whether the team has a core or a supporting task. In addition to fixed effects, all models are estimated using clustered heteroscedasticity/robust standard errors to account for potential design effects due to the nested structure of the survey by country, sector‐size combination, organisation and team.

The majority of bivariate correlations between all variables are small to moderate. The highest estimated correlation among variables in the analyses is between respondent’s year of education and co‐workers’ average years of education (corr = 0.67). Additional analyses excluding co‐worker’s education do not result in any change in model results, and there are therefore no concerns about multicollinearity affecting the findings.

## Findings

Table 2 shows the outcomes for individual performance. In these models of individual‐level performance, the team fixed effects partition out the variance attributable to team‐level factors. In our data, 79.6 per cent of the variance of performance is due to variation across workers within teams, and our full model explains 17.7 per cent of the intra‐workplace variance in individual performance.

**Table 2:** Team fixed‐effects linear regression of worker performance where employees and co‐workers are working from home

	*B*	SE
Working from home—<1 day a month^a^	−0.532***	0.14
Working from home—<1 day a week	−0.828***	0.21
Working from home—1 day a week	−0.916***	0.22
Working from home—more than 1 day a week	−0.670***	0.21
Proportion of co‐workers working from home	−2.970*	1.25
Job autonomy	0.321***	0.02
Job satisfaction	0.233***	0.04
Organisational commitment	0.188***	0.02
Job demands	−0.101***	0.03
Tenure in years	−0.065	0.07
Tenure in years squared	0.033	0.02
Permanent worker	−0.471*	0.21
Part‐time worker	0.221	0.18
Hourly wage	−0.045	0.12
Commuting time in hours	0.322	0.29
Years of education	0.019	0.04
Female	0.182	0.13
Living with minor child at home	−0.134	0.13
Living with partner	0.044	0.14
Average job autonomy—co‐workers	0.036	0.20
Average job satisfaction—co‐workers	−0.311	0.32
Average commitment—co‐workers	−0.017	0.18
Average job demands—co‐workers	−0.219	0.21
Average tenure—co‐workers	0.588	0.48
Proportion of co‐workers with permanent contract	−2.479	1.67
Proportion of co‐workers part time	0.521	1.51
Average hourly wage—co‐workers	−0.401	0.67
Average commuting time—co‐workers	1.867	2.29
Average years of education—co‐workers	−0.271	0.33
Proportion of co‐workers—female	0.139	1.01
Proportion of co‐workers with minor child	0.321	0.97
Proportion of co‐workers with partner	−0.510	1.13
Survey mode	−0.525	0.69
Constant	29.463***	8.95
Observations	8,637	
No of teams	828	

Notes

Cluster‐robust standard errors in parentheses. Analyses include work unit fixed‐effects and dummy variables for two‐digit ISCO occupations (employee) and one‐digit ISCO occupations (co‐worker). Wage and tenure variables are log‐transformed, commuting time square‐root transformed.

a Reference category: working from home never or almost never.

*** *p* < 0.001;

** *p* < 0.01;

* *p* < 0.05.

**Figure 1:**
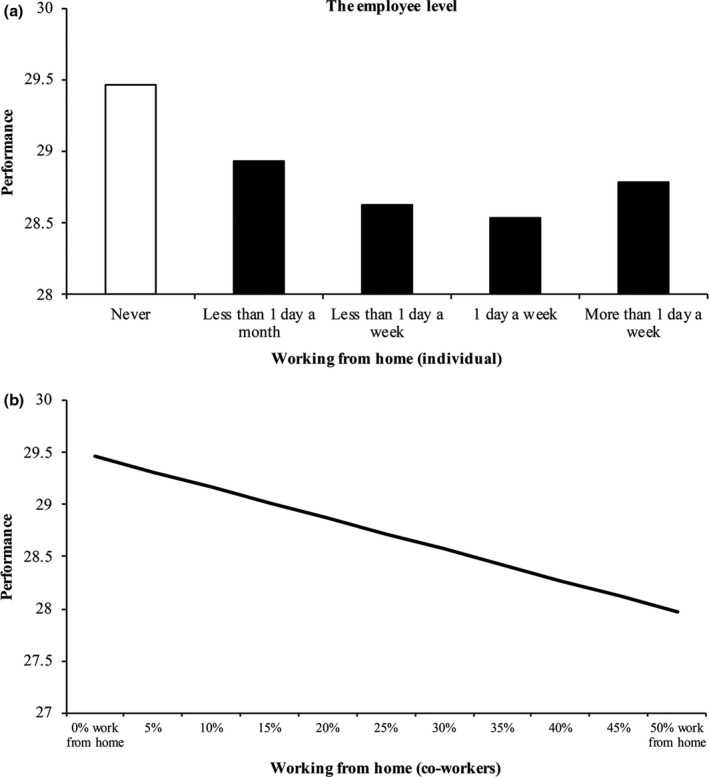
The effect of individual employee (a) and co‐workers (b) working from home on the individual employee’s work performance *Notes:* Results are based on 8,637 respondents in 828 teams and 257 establishments. Black bars show significant (*p* < 0.05, two‐sided) deviations from reference category ‘Never’. Full results are reported in Table 2. Source: ESWS.

The results show first of all that in this sample, an individual employee working from home is negatively related to the performance of that employee as compared to other workers on the team who do not work from home. Keeping all measured and unmeasured establishment‐level and team‐level factors and measured employee and job characteristics constant, working from home results in an employee performing worse. The results indicate that even a small amount of working from home, less than one day a month, negatively affects employee performance. A higher number of hours of working from home cause further negative effects on individual work performance, but this decline is only slight, up to two days a week, and these differences between high and low levels of working from home on performance are not significant. The key distinction therefore seems to be between working from home and not working from home, which is illustrated in Figure 1a. An alternative explanation is that low‐frequency teleworking may be more frequent among workers with irregular or flexible work schedules (i.e. changing shifts or working hours do not allow the worker to work from home more regularly or on fixed days of the week), and scheduling irregularity acts as a confounder because it negatively influences worker’s performance (Martens *et al.*, 1999). While our data do not allow to control for this factor, we repeated our analyses by categorising workers who work less than one day per month among those who never or almost never telework, changing the threshold definition of teleworking to a minimum of a few hours per week. The inferences about our hypotheses for own teleworking and co‐workers’ teleworking do not change after altering the definition of telework. Based on these robustness tests, we are confident that the results are not driven by factors underlying less frequent forms of telework.

To understand why individual performance deteriorates when employees work from home, we should consider the behaviour of both those employees and their co‐workers, as Table 2 shows. The more his or her co‐workers work from home, the worse the performance of the employee, which confirms Hypothesis 1. Although the explained variance is small (only 0.5 per cent of the total variance), the effect size suggests a non‐negligible impact of co‐workers working from home on individual performance: holding all other factors constant, when 50 per cent of the co‐workers of the employee work from home at least one day a month, the individual’s work performance decreases by 38 per cent of the sample standard deviation compared to no co‐workers working from home. The implied effect is illustrated in Figure 1b.

Including all independent variables and controls, the most influential factors for performance are, as expected, certain job characteristics and commitment. High levels of autonomy, satisfaction and commitment relate positively to individual performance, while perceptions of physical job demands decrease performance. Having a partner and children are not related to individual performance.

**Figure 2:**
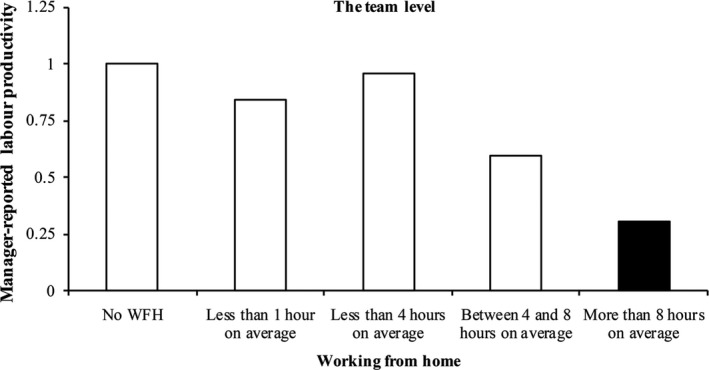
Working from home (in hours per week) and team performance as reported by the direct team manager *Notes:* Results are based on 516 teams and 153 establishments. Black bars show significant (*p* < 0.05, two‐sided) deviations from reference category ‘Never’. Full results are reported in Table 3. Source: ESWS.

After having tested the influence of working from home on individual performance, we now move to the analysis of team performance as reported by the direct manager. Table 3 shows that when team members on average spend a high number of hours working from home, a manager’s team performance rating is lower than when no team members are working from home. The impact of team‐level working from home on manager‐reported productivity is, however, non‐linear: Figure 2 suggests that direct managers prefer that employees work from home less than eight hours per week, since the team’s productivity declines at higher levels of working from home, but less frequent working from home does not harm productivity. Hypothesis 2 is thus partly confirmed. The effect of high frequency of working from home is thereby substantial: if a team were to increase working from home by eight hours or more, the likelihood of a very good performance evaluation decreases by 70 per cent as compared to teams in which no workers work from home.

**Table 3:** Establishment fixed‐effects ordered logistic regression of manager‐reported labour productivity for team‐level working from home in hours per week (WFH)

	*B*	SE
Less than 1 hour working from home team vs. no working from home team	−0.174	0.28
Less than 4 hours working from home team vs. no working from home team	−0.042	0.33
Between 4 and 8 hours working from home team vs. no working from home team	−0.518	0.36
More than 8 hours working from home team vs. no working from home team	−1.194***	0.44
Average job autonomy	0.051	0.07
Average job satisfaction	−0.035	0.15
Average organisational commitment	0.022	0.10
Average job demands	0.004	0.09
Average tenure in years	0.186	0.24
Proportion of permanent workers	−1.252	0.96
Proportion of part‐time workers	−0.642	0.66
Average hourly wage	−1.340*	0.53
Average commuting time in hours	−0.288	1.01
Average years of education	0.240*	0.11
Proportion of female workers	0.387	0.45
Proportion of workers living with minor child at home	−0.079	0.57
Proportion of workers living with partner	1.261*	0.58
Size of team (no of workers)	−0.098	0.18
Core function team (ref: supporting)	−0.152	0.23
Observations	516	
No of organisations	153	

Notes

Standard errors in parentheses. Analyses include establishment fixed effects. Wage and tenure are log‐transformed, commuting time square‐root transformed.

*** *p* < 0.001;

** *p* < 0.01;

* *p* < 0.05.

### Robustness analysis

We have asserted that working from home influences work performance, but the influence may also operate in the opposite way. To assess the reverse causality bias, we performed instrumental variable regressions (Wooldridge, 2010). Organisations and teams vary in their formal support of working from home. Knowing that such formal support exists is likely to inform employee decisions about working from home. Such knowledge is therefore likely to influence the choice of telework, but unlikely to influence someone’s performance. Knowing that working from home is supported may, however, increase workers’ organisational commitment and job satisfaction, possibly improving their performance through higher motivation. This leads to correlated error terms and biased estimates from the instrumental variable regression. However, controlled for these factors, knowledge of working from home policy should be uncorrelated with the error term of performance. We therefore include these and all other control variables in the instrumental variable estimation. Instrumental validity tests show that the IV model is identified (Anderson LM statistic (1) = 1,097.3; *p* = 0.000) and the instrument is strong (Cragg–Donald Wald *F* = 1,128.1, Stock–Yogo 10 per cent bias critical value = 16.38).

An additional problem may be the differential selection of productive workers in establishments that do and do not support working from home. We estimate a fixed‐effects instrumental variable regression model to exclude confounding variance between establishments and additionally to control for employee characteristics. We dichotomised our measure of working from home to simplify the causal effect identification. Team fixed‐effects IV and fixed‐effects regression estimates of the causal effect of working from home on task performance do not indicate endogeneity bias (see Appendix 2, Table A1).

## Conclusion and discussion

Empirical research on the influence of working from home on performance has typically focused on individual employees working from home. In this contribution, we aimed at improving our understanding of the importance of working from home on work outcomes by studying co‐workers working from home as well. Employees do not exist in a vacuum as they work. Because employees need to work together in many, if not most, workplaces, we argue that performance is not only dependent on individual employees working from home but also on the working from home of their colleagues. Using unique 2016 data relating to 11,011 employees in 869 teams in 259 work establishments in nine European countries, we show how employees and co‐workers working from home was linked to the employees’ and their teams’ performances. Our study results in three substantive findings.

First, we found that individual employees perform better when their colleagues do not work from home. The extent to which co‐workers’ work from home appears to be consequential for the functioning of the individual employee. The higher the percentage of co‐workers working from home, the worse the performance of the employee. Working together is more problematic, when employees cannot exchange critical information (Knights and McCabe, 2003). This finding shows that it is important to take into account how employees influence each other and how they use each other’s skills and knowledge, because these considerations have consequences for individual‐level performance. Our finding is in line with the literature that working from home leads to the intensification of labour (Felstead and Henseke, 2017), and helps to understand why this is the case (Kelliher and Anderson, 2009). Because co‐workers are not immediately available, it will take more effort on the part of the individual employee to make use of their skills and knowledge. Although working from home does not indicate that employees are absent, these results underline the idea that digital presence cannot really compensate for corporeal presence viewed from the co‐work perspective. This is also dependent on the nature of work (Boell, Cecez‐Kecmanovic, and Campbell, 2016) and relates to the wider literature on telework and the role of ICT.

Second, we show that manager‐reported team performance is worse when co‐workers work often from home. Our results, as reported by managers, further back up the reports concerning individual employee performance. Managers rate team productivity higher when team members work from home no more than one day per week. This underlines the idea that managers want to monitor employees, and they have more possibilities to do so when team members work less from home (Van Dyne, Kossek, and Lobel, 2007). It reflects the manager’s will to govern and illustrates that there are problems when members of the team are working from home. Control is less possible. This has particularly important consequences for how the value of other flexible work arrangements should be perceived. The traditional selling point of flexible work arrangements has been that organisations can distinguish themselves in a competitive marketplace by being better at attracting talented employees (Kossek and Thompson, 2016). However, the practical consequences of working from home illustrate that it is difficult to demonstrate a business case for flexible work arrangements (De Menezes and Kelliher, 2011) because outcomes are less profitable. In this way, team working is easy to fail when working from home (Rose and Miller, 1992). This negative influence of working from home on multiple levels of performance may explain why companies in an excellent position to encourage it, such as Yahoo!, IBM, Bank of America, HP and Best Buy, actually discourage it. The existence of direct managers who are not very positive about their team members working frequently from home might be a source of pressure on top managers to drop their support for the practice. Some top managers have even discontinued their companies’ telecommuting programs (Guynn, 2013; Weinert *et al.*, 2014).

Third, working from home was negatively related to individual employee performance in our data set of multiple work establishments. This is in line with the finding that social and professional isolation is the result (Kurland and Bailey, 1999; Crandall and Gao, 2005), which overall leads to less performance. However, most findings in the literature are more often positive than negative (Allen *et al.*, 2015). One reason for this difference could be that we deliberately focused on employees in their organisational context, while previous research often considered employees in general without considering this context or analysed workers in only one organisation. This also depends on the outcomes studied. Work–life balance and performance are different outcomes. We demonstrated a negative influence across a diverse set of sectors and countries. Our data imply that the goal of building common ground through engaging in sociality may be problematic for teleworkers, given their reported need to demonstrate that they are working hard (Golden, 2006). Moreover, being away from the office makes them invisible, raising fears that they will miss out on opportunities for promotion, rewards and positive performance reviews (Cooper and Kurland, 2002).

It is possible that our finding of negative effects is dependent on certain boundary conditions, most notably perhaps the use of IT‐enabled online platforms facilitating cooperation and knowledge sharing, which may help optimise the performance of teams on which many workers are working from home. While an analysis of the impact of such technologies is beyond the limits of our paper, they present issues of considerable interest because the benefits of IT‐enabled technologies are much‐debated: Do employees perceive these information technologies as useful tools facilitating their interactions with co‐workers and managers and their work in general, or rather, as an attempt by the organisation to increase surveillance of and control over their work? We encourage future research on working from home to incorporate IT and how it is perceived by the workforce into the analyses performed.

Our results leave several questions unanswered, such as what actually happens when employees work from home, including how they interact with employees at the office and with each other (Vayre and Pignault, 2014). In this paper, we focused deliberately on co‐workers working from home as a first step towards introducing the co‐worker as an important understudied factor in this type of research. We encourage other researchers to focus more on the behaviour of co‐workers in understanding the behaviour of individual employees and to study what exactly mediates the effect of co‐workers working from home on individual and team performance. This might also clarify the divergences and paradoxes often found in the current literature on working from home. It might have to do with the nature of the work. When organisational practices are taken into account in the previous literature, it is often restricted to the level of the whole organisation via various kinds of company policies (Martínez Sánchez, Pérez, De Luis Carnicer, and Vela Jiménez, 2007) or to the level of the direct manager. Both of these influence the behaviour of the employee, but the co‐worker is as yet an understudied factor. Furthermore, our research was based on the assumption that working from home is voluntary, thus providing employees with greater flexibility in choosing the location of their work (Duxbury, Higgins, and Neufeld, 1998). But what if it is not voluntary? Lapierre *et al. *(2016) show that non‐voluntary working from home is associated with negative outcomes such as strain resulting from work–family conflict. It would be valuable to conduct research that takes into account the fact that some employees work voluntarily from home while others do not, and the effect this distinction has on performance. Moreover, it would be interesting to gain new data to differentiate between all‐day versus part‐of‐the‐day (e.g. a few hours) teleworking. If people are gone for the entire day, it might be more detrimental for work performance than if they are only out of the office a few hours. Another drawback of this study is its cross‐sectional nature; although we performed additional instrumental variable analyses concluding that it is more likely that working from home has an effect on performance instead of the other way around, the prevalence of cross‐sectional designs is a common problem in the field (Allen *et al.*, 2015). It would therefore be beneficial to obtain longitudinal data from employees within an organisation to see whether the relationship found is stable over time.

Given the possibilities now available because of IT for both employees and managers, we are not inclined to suggest that employers should encourage employees to work from the office instead of from home and that employees should not work from home anymore. Society has to deal with new technological possibilities, and governments should think about (informal) rules to accommodate both employees and employers. This is all the more important given that a social‐economic divide is looming regarding new technologies. Not everybody is able to profit from working from home and other flexible work arrangements. With respect to employees, we advise to take into account what their colleagues are doing. Working together is also a social event and an optimum needs to find how much working from home is desirable and possible given both positive and negative outcomes. Training might be a helpful tool to learn how to deal with colleagues working from home. We advise organisations to focus on improving cooperation among team members when many employees work from home. Interaction with colleagues, whether or not someone is working from home, is the key to cooperation and efficiency, and it is a challenge to construct teams in which teleworkers work efficiently. At this moment, organisational policies related to working from home mostly focus on the level of the individual worker (Butts, Casper, and Yang, 2013); our paper instead shows that the composition of the team and how many of its members are working from home should also be taken into account. The general lesson to be learned from our research is that organisational surveys with nested designs that allow the investigation of co‐worker effects are important for better understanding the impact of flexible work arrangements.

## Appendix 1

**Table:**

Country	%
UK	0.07
Germany	0.08
Finland	0.08
Sweden	0.10
Netherlands	0.24
Portugal	0.11
Spain	0.08
Hungary	0.13
Bulgaria	0.13
*Industry*	
Manufacturing	0.23
Health care	0.23
Higher education	0.17
Transport	0.14
Financial services	0.13
Telecommunication	0.10

Notes

Based on 8,637 respondents in 828 teams and 257 organisations.

## Appendix 2

**Table A1:** Instrumental variable estimation of the individual‐level effect of working from home (WFH)

	*B*	SE	*T*	*p*
Fixed‐effects IV model	−1.21	0.31	−3.92	0.000
Fixed‐effects model	−0.53	0.11	−4.38	0.000

Notes

*N* = 8,637. Models include all controls in the main individual‐level analyses, excluding co‐worker effects. WFH measure dichotomised. Instrument: dichotomised knowledge about formal and informal organisational support of WFH (answer categories: formal support and informal support = 1, don’t know about support and no support = 0).
